# Neuroendocrine regulation of appetitive ingestive behavior

**DOI:** 10.3389/fnins.2013.00213

**Published:** 2013-11-15

**Authors:** Erin Keen-Rhinehart, Katelynn Ondek, Jill E. Schneider

**Affiliations:** ^1^Department of Biology, Susquehanna UniversitySelinsgrove, PA, USA; ^2^Department of Biological Sciences, Lehigh UniversityBethlehem, PA, USA

**Keywords:** food hoarding, leptin, ghrelin, neuropeptide Y, maternal effects, gestational programming, hypothalamus, obesity

## Abstract

Food availability in nature is often irregular, and famine is commonplace. Increased motivation to engage in ingestive behaviors increases the chance of survival, providing additional potential opportunities for reproduction. Because of the advantages conferred by entraining ingestive behavior to environmental conditions, neuroendocrine mechanisms regulating the motivation to acquire and ingest food have evolved to be responsive to exogenous (i.e., food stored for future consumption) and endogenous (i.e., body fat stores) fuel availability. Motivated behaviors like eating occur in two phases. The appetitive phase brings animals into contact with food (e.g., foraging, food hoarding), and the more reflexive consummatory phase results in ingestion (e.g., chewing, swallowing). Quantifiable appetitive behaviors are part of the natural ingestive behavioral repertoire of species such as hamsters and humans. This review summarizes current knowledge about neuroendocrine regulators of ingestive behavior, with an emphasis appetitive behavior. We will discuss hormonal regulators of appetitive ingestive behaviors, including the orexigenic hormone ghrelin, which potently stimulates foraging and food hoarding in Siberian hamsters. This section includes a discussion of the hormone leptin, its relation to endogenous fat stores, and its role in food deprivation-induced increases in appetitive ingestive behaviors. Next, we discuss how hormonal regulators interact with neurotransmitters involved in the regulation of ingestive behaviors, such as neuropeptide Y (NPY), agouti-related protein (AgRP) and α-melanocyte stimulating hormone (α-MSH), to regulate ingestive behavior. Finally, we discuss the potential impact that perinatal nutrient availability can have on the neuroendocrine regulation of ingestive behavior. Understanding the hormonal mechanisms that connect metabolic fuel availability to central appetite regulatory circuits should provide a better understanding of the neuroendocrine regulation of the motivation to engage in ingestive behavior.

## Introduction

Although obesity was rare in the human population for thousands of years (Haslam, 2007), it is now one of the leading health issues worldwide and has been declared a global epidemic by the World Health Organization (Caballero, 2007). Despite concerted efforts to solve this problem, obesity rates continue to rise worldwide. To understand human ingestive behavior and the origins of this epidemic, it is useful to consider an evolutionary perspective (Bronson, 1989; Neel, 1999; Prentice, 2005; Schneider, 2006). Fluctuating food supply and famine have been a consistent threat to survival for most animals, including humans (Prentice, 2005). Selection pressures resulting from these fluctuations favor the ability to store energy sources on the body as fat and/or the propensity to store food in the home. These abilities provide a buffer against a variable or unpredictable food supply, and thus allow animals to optimize reproductive success (Prentice, 2005; Schneider, 2006). These phenotypic properties are physiologic (turning off unnecessary functions), metabolic (slowing basal metabolic rate), behavioral (binge eating, food hoarding, decreased physical activity), and adipogenic (generation of internal fat stores, (Prentice, 2005). In western, industrialized societies, humans inhabit an environment with an overabundance of highly palatable, easily accessible food sources, transforming these once beneficial adaptations into health liabilities. Therefore, understanding the basic neuroendocrine regulation of food and body fat storage and the mechanisms underlying the adaptations to a food-insecure environment is a central issue to understanding and combating the obesity epidemic (Ulijaszek, 2002).

Human obesity studies typically focus on metabolism, adipogenesis, and meal patterns. Human foraging and hoarding behavior typically is dismissed as irrelevant to understanding the health issues surrounding overeating, obesity, and diabetes. Some data is available, however, on food purchasing behavior, and these studies indicated that 85% of the energy content of the human diet consists of food purchased for consumption at home (Ransley et al., 2003). These types of studies also confirm the well-known anecdote that purchasing food when food deprived or “hungry” increases food purchasing, (Dodd et al., 1977; Tom, 1983). In addition, obese individuals are likely to purchase more food per person and more calorically dense food (Ransley et al., 2003; Epstein et al., 2007). After foraging for food at the grocery store, humans then store the food at home for future consumption in freezers and pantries (a.k.a. hoarding). Efforts to understand the neuroendocrine differences responsible for these incongruities between the behaviors of lean and obese individuals have employed a variety of animal models.

Food intake is the most studied ingestive behavior, but other ingestive behaviors are likely to be important. In the long history of research in behavioral endocrinology, motivated behavior is thought to be comprised of two main phases: the appetitive and the consummatory phases (Craig, 1918). Appetitive behaviors are flexible, non-stereotyped responses that bring an animal into contact with a goal object, whereas consummatory behaviors are the final reflexive, stereotyped responses after decisions and efforts have been made to reach the goal object. Both appetitive and consummatory behaviors can increase with hunger and the desire immediately to consume food and to store energy. Eaten food can be stored on the body as adipose tissue depots, while hoarded food is stored in the home or burrow for later consumption during energetic deficits. The important distinction lies in the fact that consummatory behaviors also reflect the ability to perform motor actions. Therefore, measures of food intake can be confounded by limits on the ability of the animal to eat or digest food, or by many other postingestional cues. By contrast, appetitive behaviors are a more “pure” reflection of motivation for acquiring fuels either for eating or calorie storage. Some appetitive ingestive behaviors occur at different times than consummatory behaviors, and appetitive behaviors most useful for scientific study are those that can occur even in the absence of changes in food intake (Bartness and Clein, 1994; Bartness, 1997; Buckley and Schneider, 2003). For example, hungrier animals initiate meals sooner, avidly consume an unpalatable substance, make more effort to gain access to food, and hoard more food in their home or burrow (reviewed by Keen-Rhinehart et al., 2010; Bartness et al., 2011; Schneider et al., 2013). This review will focus on appetitive ingestive behaviors that: (1) are easily quantified, (2) occur separate in time and space from consummatory behaviors, (3) are controlled by different chemical messengers, neural substrates, and environmental stimuli than consummatory behaviors, and (4) are able to provide an indication of the animals' internal motivational state. The neuroendocrine mechanisms that control consummatory ingestive behavior (food consumption) are relatively well studied (for reviews see: Kalra et al., 1999; Abizaid and Horvath, 2012; Kageyama et al., 2012). After a brief review of the neuroendocrine regulation of consummatory ingestive behavior, this review will focus mostly on the neuroendocrine basis for appetitive behavior, with a major emphasis on food hoarding as a quintessential appetitive behavior.

## Neuroendocrine regulation of consummatory ingestive behavior

Historically, the majority of ingestive behavior studies have used laboratory rats or mice as model systems, measuring the food consumption of animals housed in isolation. Therefore, it is useful to review these data as a starting point for understanding the neuroendocrine control of ingestive behavior. Many neuroendocrine factors affect food intake in laboratory animals (reviewed by Schneider and Watts, 2002; Schneider et al., 2012). Adrenal steroids (Stevenson and Franklin, 1970), and other proteins and peptides of the hypothalamic-pituitary-adrenal (HPA) axis significantly impact food intake (reviewed by Heinrichs and Richard, 1999; Bergonzelli et al., 2001). In addition, estradiol, testosterone, progesterone, and other neuroendocrine factors of the hypothalamic-pituitary-gonadal axis (HPG) axis affect food intake (reviewed by Wade, 1976; Schneider and Wade, 1999; Schneider et al., 2002). Finally, the metabolic hormones, leptin, insulin, glucagon, ghrelin, and glucagon-like peptide-I (GLP-I), as well as central factors, such as neuropeptide Y (NPY), agouti-related protein (AgRP), cocaine and amphetamine-regulated transcript (CART) and the melanocortins reliably affect consummatory ingestive behavior in laboratory animals (reviewed by Barsh and Schwartz, 2002; Zigman and Elmquist, 2003; Schwartz and Porte, 2005). Most recently, there has also been a push to understand the role of reward circuits and dopamine in the regulation of ingestive behavior (Abizaid et al., 2006b; Hommel et al., 2006; Abizaid, 2009). We shall attempt to review the contributions of each of these three systems to the neuroendocrine regulation of consummatory ingestive behavior.

### The arcuate perspective on the regulation of consummatory ingestive behavior

The often co-localized orexigenic neuropeptides AgRP and NPY are the major products of the Arc that stimulate food intake (Kalra et al., 1999; Morton and Schwartz, 2001; Ellacott and Cone, 2004). The anorexigenic neuropeptide α-melanocyte stimulating hormone (α-MSH), a cleavage product of the proopiomelanocortin (POMC) gene, and CART are thought to oppose the effects of NPY and AgRP (reviewed by Zigman and Elmquist, 2003; Schwartz and Porte, 2005). According to the Arc perspective, anorexigenic and orexigenic neuron populations in the Arc respond to changes in the availability of oxidizable metabolic fuels, body fat stores, and/or peripheral hormones such as glucocorticoids, ghrelin, and leptin (Baskin et al., 1999; Cone et al., 2001). Peripheral hormones bind to receptors on Arc NPY/AgRP and POMC/CART neurons, affecting gene transcription (Coppari et al., 2005; Elmquist et al., 2005) and the synaptic inputs to the Arc neurons (reviewed by Zigman and Elmquist, 2003; Gao et al., 2007; Gyengesi et al., 2010; Briggs and Andrews, 2011; Eckel, 2011).

Although the plethora of evidence in support of the “arcuate perspective” seems to provide a simple, well-constructed model for the regulation of food intake at first glance, many other studies indicate that the Arc is neither necessary nor sufficient for the regulation of ingestive behavior (Grill, 2010). For example, neonatal Arc ablation does not disrupt food intake (Coppari et al., 2005), nor does adult Arc ablation have an effect if the animals are given GABA agonists in the parabrachial nucleus [PBN (Wu et al., 2009)]. In addition, food intake increases when the leptin receptor (LepR) is deleted outside the Arc and when orexigenic neuropeptides are injected in extra-arcuate regions of the brain (Faulconbridge et al., 2005; Dhillon et al., 2006; Musatov et al., 2007). The “arcuate perspective” also fails to incorporate extra-Arc populations of NPY, AgRP, and α-MSH neurons in areas such as the dorsomedial hypothalamus (DMH), ventromedial hypothalamus (VMH), paraventricular nucleus of the hypothalamus (PVN) and lateral hypothalamus (LHA), as well as how these cells are connected to the PBN, the dorsomotor nucleus of the vagus (DMV) and the nucleus of the solitary tract (NTS) in the hindbrain, that are responsible for the motor movements necessary for food intake (Zheng et al., 2010). It has also been demonstrated that peripheral sensory information reaches the diencephalon via the afferent visceral-central pathway and that vagal afferent NTS neurons connecting directly to pre-oral motor neurons of the reticular formation are both essential and sufficient for consummatory ingestive behavior (reviewed by Grill, 2010). Therefore, examining the role of these extra-Arc areas in the regulation of consummatory ingestive behavior in the future should provide a more complete understanding of the neural circuitry controlling food intake.

### Role of the HPA axis in the regulation of consummatory ingestive behavior

It has long been known that stress and adrenal steroids affect food consumption (Stevenson and Franklin, 1970). The HPA axis provides critical input to the neuroendocrine mechanisms that regulate energy intake, storage, and expenditure (reviewed by Heinrichs and Richard, 1999). Within the HPA axis, corticotropin-releasing hormone (CRH) regulates the secretion of adrenocorticotropic hormone (ACTH), which controls the secretion of adrenal steroids. Food deprivation decreases CRH expression in the PVN (Brady et al., 1990). CRH plays an anorexigenic role in the basal regulation of food intake (Karolyi et al., 1999). Although CRH is anorectic, glucocorticoids are orexigenic, and obesity does not occur in their absence. For example, lesion-, diet-, NPY-, and leptin gene mutation-induced obesity are reversed by adrenalectomy or by lack of glucocorticoids caused by shutting down the HPA axis (Bruce et al., 1982; Romsos et al., 1987; Stanley et al., 1989; Bray et al., 1992; Feldkircher et al., 1996).

HPA axis hormones and neuropeptides are highly conserved across a wide array of species, with regard to both chemical structure and the effects on the ingestive behavior (Landys et al., 2006). The CRH gene sequence is highly homologous in organisms from fish to mammals, and central CRH treatment decreases food intake in all classes of vertebrates tested to date (Carr, 2002) without increasing plasma glucocorticoids, unlike peripheral administration that increases both glucocorticoid levels and food consumption (Glowa et al., 1992). Although CRH is anorexigenic, glucocorticoids typically stimulate food consumption (Dallman et al., 1993, 2004) and provide negative feedback to inhibit CRH production and release. Thus, orexigenic effects of glucocorticoids could occur via direct effects on appetite regulatory mechanisms or via indirect effects by inhibiting the synthesis and release of CRH (reviewed by Heinrichs and Richard, 1999). The opposing effects of CRH and glucocorticoids are likely part of an adaptive response to stress (Sapolsky et al., 2000). Stress causes the release of adrenaline and CRH, which inhibit ingestive behaviors and trigger “fight-or-flight” behaviors, increasing the likelihood of survival. Increases in CRH directly stimulate sympathetic tone and the uptake of oxygen and metabolic fuel by the brain and muscles. The resulting rise in glucocorticoids inhibits CRH via action on central receptors to turn off the stress response, and then ingestive behavior resumes once the stressful situation has been resolved (Sapolsky et al., 2000). This sequence of events facilitates the reinstatement of energy balance by replacing the energy expended on the fight-or-flight response (Sapolsky et al., 2000). Stress responses are energetically expensive, and the ability to replenish lost energy is affected by fluctuating variables such as food availability, mate availability, and ambient temperature making the metabolic fuel availability a significant modulator of the effects of CRH and glucocorticoids on behavior.

### Role of reward circuits in the regulation of consummatory ingestive behavior

Further complexity in the neuroendocrine regulation of consummatory ingestive behavior is afforded by the involvement of the mesolimbocortical dopaminergic (DA) system, which includes the nucleus accumbens, ventral tegmental area (VTA), and parts of the amygdala. This system is implicated in modulating behaviors such as learning, appetite, motivation and reward, as well as the willingness to work to access highly palatable foods (reviewed by Abizaid, 2009). Partly because of the overabundance of data supporting the hypothesis that obesity can be attributed to an addiction to food (Stice et al., 2012; Volkow et al., 2013), numerous studies have been conducted to investigate the connections between metabolic hormones and central reward circuits (for review see Hirasawa et al., 2007; Pandit et al., 2011; Schellekens et al., 2012; Volkow et al., 2013). For example, leptin is apparently able to reduce appetite by diminishing the rewarding value of food. Studies supporting this notion show that increased leptin signaling specifically in the VTA decreases food intake and firing rates of DA neurons while treatments that impair leptin signaling in the VTA increase DA neuronal firing rates, food intake and the proclivity to ingest highly palatable foods (Fulton et al., 2002, 2006; Hommel et al., 2006; Abizaid, 2009). Ghrelin also appears to modulate food intake via action in the VTA, and peripheral ghrelin treatment increases DA turnover, food intake and synapses connecting with DA cells in the VTA in mice (Abizaid et al., 2006a,b). Therefore, the midbrain has emerged as an important site for the integration of information about peripheral metabolic factors in the service of ingestive behavior regulation.

## Appetitive ingestive behavior

It is typically assumed that if a substance applied to the brain affects food intake, it must function in the service of energy balance. One must also consider the possibility that these factors may have altogether different purposes for an animal in a naturalistic setting. It is unlikely that these other functions will be observed in laboratory experiments studying singly housed animals with unlimited food and few prospects for species-typical behaviors or opportunities for energy expenditure. For example, experimentally induced increases in food consumption in an individually housed animal may actually affect motivation that would stimulate foraging, hoarding, or hunting behavior in a more naturalistic setting. Experimentally induced decreases in hunger might increase mate searching, courtship, mating, nest building, incubation, migration, or hibernation. In nature, ingestive behavior must be balanced with other priorities such as predator avoidance, food availability, and copulation. Regardless of the animal model, experiments examining only consummatory ingestive behavior in isolated animals might support the idea that 40 or more redundant peptide systems regulate this one variable, food intake. Therefore, manipulations of environmentally relevant variables (e.g., nutrient availability) to determine how these affect the expression of appetitive behaviors (those other than food intake) are necessary to provide a more complete understanding about the neuroendocrine regulation of the motivation to eat.

### Appetitive behaviors: indicators of motivation

Ingestive behavior is multifaceted and cannot be accurately captured by measuring the amount of food consumed because swallowing is only one step in a long sequence of behaviors necessary for eating. Appetitive ingestive behaviors aim to ensure that sufficient energy will be available for future energetic needs (Craig, 1918; Everitt et al., 1984). Studying appetitive ingestive behaviors is the preeminent way to assess the internal motivation to consume food. Furthermore, the study of appetitive behaviors can provide information about how environmental factors such as nutrient availability impact behavioral priorities.

In certain well-designed experimental paradigms, appetitive behaviors are separable from consummatory behaviors, and the mechanisms underlying these two phases of ingestive behaviors can be studied separately. The significance of the separation of the appetitive and consummatory phases of ingestive behavior lies in the fact that the two phases are initiated by different environmental conditions via various neuroendocrine factors that act on separate neural substrates (reviewed by Ball and Balthazart, 2008). One of the clearest examples of the separate neuroendocrine regulation of these behavioral phases is the control of consummatory but not ingestive behaviors by the brain stem (Grill and Smith, 1988; Grill and Kaplan, 2001, 2002). This is skillfully illustrated in experiments showing that decerebrate rats have intact neuroendocrine regulation of intraoral food intake in the absence of the ability to express any appetitive behaviors (Grill and Kaplan, 1992, 2001, 2002; Grill and Hayes, 2009).

To understand the neuroendocrine mechanisms regulating appetitive ingestive behaviors and thus the motivation to eat, it is necessary to look beyond the commonly studied rats and mice to other animal models where appetitive behaviors such as food hoarding are separable from food consumption. While rats and mice typically increase food intake in response to fasting or food restriction (Ross and Smith, 1953; Hill et al., 1983, 1984), hamsters, like humans, reliably increase food hoarding instead of food intake in response to energetic challenges (Silverman and Zucker, 1976; Rowland, 1982; Billington et al., 1984; Bartness and Clein, 1994; Bartness et al., 1995). In fact, *Homo sapiens*, unlike rats and mice, do not typically overeat in response to food deprivation (reviewed by Bartness et al., 2011). People do, however, increase grocery shopping (foraging) and bring more food home (hoarding) in correlation with the length of time since their last meal (Dodd et al., 1977; Beneke and Davis, 1985; Mela et al., 1996). In addition, recent studies on overweight and obese children indicate that a significant proportion of them exhibit disturbances in eating behaviors that include sneaking, hiding and hoarding large quantities of food (Sonneville et al., 2013). Therefore, using a hamster model that responds to energetic challenges such as food deprivation with prolonged increases in food hoarding but not food intake permits the disentanglement of the mechanisms underlying appetitive and consummatory ingestive behaviors that are inextricably intertwined in other rodent models (for review see: Keen-Rhinehart et al., 2010; Bartness et al., 2011). Over the past two decades, studies using Siberian hamsters have thoroughly and systematically determined how appetitive ingestive behaviors are affected by fasting, diet dilution and lipectomy as well as treatment with orexigenic factors such as NPY, Y1R agonists, AgRP, and ghrelin and anorexigenic factors such as leptin, MT-II, CCK, and Y1R antagonists (Bartness and Clein, 1994; Bartness et al., 2011; Wood and Bartness, 1996a,b; Bartness, 1997; Day and Bartness, 2003, 2004; Day et al., 2005; Keen-Rhinehart and Bartness, 2005, 2007; Keen-Rhinehart et al., 2010; Teubner and Bartness, 2010; Teubner et al., 2012).

### Appetitive behavior regulation: beyond the arcuate perspective

As mentioned earlier, many investigators in the field of ingestive behavior consider the Arc to be the hub for the regulation of food intake. Therefore, initial experiments regarding the neuroendocrine mechanisms that regulate appetitive ingestive behaviors focused on this area as a starting point. As mentioned previously, food deprivation in Siberian hamsters causes large increases in food hoarding with little to no effects on food intake (Bartness and Clein, 1994; Bartness, 1997). The orexigenic hormone ghrelin is increased in response to food deprivation in hamsters, similarly to other animals (Ariyasu et al., 2001; Asakawa et al., 2001; Keen-Rhinehart and Bartness, 2005). When hamsters are treated with ghrelin at doses that produce circulating ghrelin concentrations similar to a 48 h fast, there is an initial, small increase in food intake followed by a massive, long-term increase in food hoarding behavior (Keen-Rhinehart and Bartness, 2005). We now know that both food deprivation and ghrelin stimulate Arc NPY and AgRP neurons (Hahn et al., 1998; Mercer et al., 2000), triggering the release of these orexigenic neuropeptides (Chen et al., 2004). As one would expect, central treatment with either of these orexigenic factors trigger marked increases in food hoarding with minimal increases in food intake [i.e., NPY (Day et al., 2005; Keen-Rhinehart and Bartness, 2005) and AgRP (Day and Bartness, 2004)]. Conversely, the anorexigenic hormone leptin is postulated to act on LepR-expressing hypothalamic neurons (Baskin et al., 1999; Elmquist, 2001) to reduce appetite by reducing expression of NPY and AgRP, and by increasing expression of anorexigenic neuropeptides like α-MSH (Baskin et al., 2001; Elmquist et al., 2005). In hamsters, central or peripheral treatment with leptin reduces fasting- and ghrelin-induced increases in food hoarding behavior (Keen-Rhinehart and Bartness, 2008).

Because the expression of appetitive and consummatory ingestive behaviors are separable in hamsters, the neural substrates controlling food hoarding should be at least partially distinct from those that govern food intake. Several studies provide data in support of that hypothesis. For example, in Siberian hamsters, Arc lesions diminish NPY-induced increases in food intake but not food hoarding (Dailey and Bartness, 2010), and studies using Y1R- and Y5R-specific agonists indicate that NPY increases food intake via action at the Y5 receptor but affects food hoarding by acting on the Y1 receptor (Keen-Rhinehart and Bartness, 2007). In addition, recent studies show that the pharmacological blockade of ghrelin activity attenuates, but cannot completely block, food deprivation-induced increases in food hoarding (Teubner and Bartness, 2013; Teubner et al., 2013).

While Siberian hamsters typically exhibit increases in food hoarding rather than food intake in response to energetic challenges, there is initially a small increase in food intake before the large increase in food hoarding behavior. Exploiting the fact that increases in food intake appear more rapidly than increases in food hoarding, Teubner et al., differentiated the brain areas activated concurrently with short-term increases in consummatory behavior from those activated later in conjunction with long-term increases in appetitive behavior (Teubner et al., 2012). Combined central NPY + AgRP injection was associated with increased food intake and neuronal activation of the PVN and perifornical area (pFA) within 1 h post-injection, but increased food hoarding and neuronal activation in the PVN, pFA, central nucleus of the amygdala (CeA) and the subzona incerta (SZI) were not detectible until 4–14 h post-injection. These data pinpoint two novel regulatory brain areas (CeA and SZI) that affect ingestive behavior. Despite the prevailing view that neurons in the Arc are critical for ingestive behavior, NPY + AgRP co-injection did not activate Arc neurons at any time when increased food hoarding was observed.

This study also demonstrated that co-injection of sub-threshold doses of NPY and AgRP increases food hoarding in Siberian hamsters, suggesting that there is a significant intercellular interaction between these two neuropeptides occurring as part of the regulation of food hoarding. Because AgRP is known to cause long-term increases in food hoarding, AgRP may be providing synergistic effects on NPY-induced food hoarding in the CeA and SZI (Teubner et al., 2012), brain areas with Y1 as well as MC4 receptors (Lyons and Thiele, 2010).

### Role of the hpa axis in the regulation of appetitive ingestive behaviors

A great deal of research has been conducted on the effects of CRH on consummatory ingestive behavior, all suggesting that CRH is anorexigenic. Comparatively little has been done to determine how CRH or other HPA axis factors affect appetitive ingestive behaviors. Food deprivation decreases hypothalamic CRH mRNA expression in several animal species (Brady et al., 1990; Glowa et al., 1992). In studies in which hunger motivation is taken as the body weight loss that produces significant increases in food hoarding, hunger motivation is decreased by adrenalectomy and CRH treatment and increased by low doses of glucocorticoids, fasting, and low temperatures (Fantino and Cabanac, 1984; Cabanac and Richard, 1995; Cabanac et al., 1997; Gosselin and Cabanac, 1997; Michel and Cabanac, 1999). Studies in rats and primates seem to indicate that CRH mediates a shift away from ingestive behaviors, affecting appetitive behaviors at lower doses than those at which food intake is impacted. In addition, studies in anuran amphibians (toads) show that CRH treatment inhibits the appetitive behavior of foraging by impairing the efficiency of visual stimuli to stimulate prey-catching behavior (Carr et al., 2002). This might be adaptive if, in the presence of a predator, the hypothalamic stress response prevents the toads from engaging in appetitive ingestive behaviors that would make them an easy target. There is also some indication that the ability of leptin to prevent fasting-induced increases in ingestive behavior is due in part to activation of central CRH circuits (van Dijk et al., 1997). Although the connection between the HPA axis and appetitive ingestive behaviors has not been studied extensively in the hamster model of food hoarding, such studies are likely to yield a great deal of useful information on the nexus between stress and ingestive behavior.

### Reward circuits and the regulation of appetitive ingestive behavior

Food intake is a motivated behavior, and animals find food, especially highly palatable, calorically dense food, rewarding. Therefore, when examining the motivation to engage in ingestive behaviors, it is necessary to consider how reward circuits influence the expression of appetitive ingestive behaviors. Studies in Mongolian gerbils (*Meriones unguiculatus*) show that the act of hoarding food increases the activation of tyrosine hydroxylase (TH)-containing cells in the nucleus accumbens, VTA, and LHA (Yang et al., 2011). TH is the rate-limiting enzyme in the chemical process of dopamine synthesis. In addition, both Mongolian gerbils and Brandt's voles (*Lasiopodomys brandtii*) that are consistent food hoarders exhibit food deprivation-induced increases in food hoarding along with cellular activation in the VTA (Yang et al., 2011; Zhang et al., 2011). The association between the activation of DA neurons in the VTA with food hoarding suggests that animals with the propensity to hoard food or with previous food hoarding experience are likely to find food hoarding especially rewarding or reinforcing. Obviously, this, like the connection between the HPA axis and ingestive behavior regulation, is greatly in need of further investigation.

## Gestational programming of ingestive behavior

The relatively recent, rapid increase in the prevalence of obesity has been attributed by some to environmental factors, including the availability of highly palatable foods and “modern” lifestyles involving less physical work, all of which have created an “obesogenic” environment. It is also possible that characteristics of the prenatal environment initiate organizational effects of hormones that modulate neuroendocrine development to produce optimal adaptation of energy balance and ingestive behavior to the anticipated postnatal environment. Several different synonyms are used to describe the process by which changes in the mother's environment lead to changes in fetal development that have permanent effects in the offspring: gestational, fetal, or maternal programming, or metabolic imprinting. Examples of factors in the mother that influence metabolism in offspring include maternal food restriction, caloric, fat, carbohydrate or protein content of food, non-nutritional stress, and nutritional status of the mother (reviewed by Armitage et al., 2004). In *Homo sapiens*, prenatal undernutrition leads to low birth weight (LBW) babies who are prone to develop metabolic syndrome, insulin resistance, and obesity as adults (Hales and Barker, 2001; Cripps et al., 2005). This relationship between LBW and later development of obesity and metabolic syndrome has been replicated in many diverse populations around the world (Hales and Barker, 2001; Gluckman et al., 2005). In addition, rapid neonatal catch-up growth caused by excessive appetite during the neonatal period acts as an additional risk factor that further increases the risk of obesity (Hales and Barker, 2001; Gluckman et al., 2005; Vickers, 2007; Vickers et al., 2007). Because formula-fed infants typically have an accelerated initial growth rate, formula feeding also increases the risk for becoming obese or overweight (Baird et al., 2005; Harder et al., 2005; Vickers, 2007; Vickers et al., 2007). The relationship between LBW and the later development of obesity and metabolic syndrome has been replicated animal models such as the rat, mouse, pig, sheep and guinea pig (for review see Hales and Barker, 2001; Cripps et al., 2005; Gluckman et al., 2005). Interestingly, maternal obesity during pregnancy and gestational diabetes can also lead to offspring obesity (Muhlhausler, 2007; Shankar et al., 2008).

From an ecological point of view, these maternal programming effects might be adaptive (that is, they might improve offspring survival and reproductive success) or maladaptive. One hypothesis is that changes in maternal energy status generate signals *in utero* to communicate reduced energy availability. These signals purportedly prepare the offspring for a postnatal environment with a low or unpredictable food supply. According to this idea, the maternal environment organizes energy balance and the central appetite regulatory circuits so that the offspring will develop traits that will allow them to cope with that particular environment (Gonzalez-Bulnes and Ovilo, 2012). A commonly reported phenomenon in mice, rats, sheep and primates is that LBW offspring have a propensity toward hyperphagia, efficient metabolism, and an increased inclination to store energy on the body as body fat (Cripps et al., 2005). These same animals might be better adapted to survive in an environment where food supply is low or unpredictable. In an environment of energy abundance, however, they are likely to develop metabolic syndrome and obesity.

### Gestational programming of consummatory ingestive behavior

As mentioned previously, impaired prenatal nutrition causes offspring hyperphagia and obesity. Given the pervasive nature of the “Arcuate perspective” on the control of ingestive behavior, most studies on the gestational programming of appetite have focused on the effects of prenatal nutrient availability on orexigenic (i.e., ghrelin, NPY, and AgRP) and anorexigenic (leptin and α-MSH) neuroendocrine factors. For instance, the stomach-derived orexigenic hormone ghrelin is elevated at birth in LBW offspring (Wang et al., 2007; Sahin et al., 2012) and continues to be elevated into adulthood (Sahin et al., 2012). LBW offspring typically exhibit rapid catch-up growth facilitated by enhanced appetite shortly after birth (Coupe et al., 2010; Breton, 2013). Therefore, increased postnatal ghrelin likely plays a role in the rapid catch-up growth observed in most models of gestational programming. In rodents, 50% maternal food restriction during the second half of gestation results in LBW pups that, like human LBW infants, have significantly increased ghrelin expression (Cortelazzi et al., 2003; Nagata et al., 2011). These animals develop hyperphagia and obesity and demonstrate that catch-up growth among LBW offspring results in programmed elevation of plasma ghrelin levels (reviewed by Breton, 2013). Arc NPY and AgRP neurons in hypothalamic slices from adult nutrient-restricted offspring are more sensitive to the stimulatory effects of ghrelin, which could indicate how LBW offspring maintain elevated expression of ingestive behavior throughout their lifespan (Yousheng et al., 2008). Together, these results suggest that appetite regulatory circuits can be gestationally programmed to favor hyperphagia and storage of energy as fat by developmental conditions that produce LBW offspring.

One of the hypothesized mechanisms for long-term changes in the neuroendocrine regulation of ingestive behavior is an alteration in hormonal milieu at critical periods of development. As mentioned above, circulating ghrelin concentrations are elevated in LBW offspring. In addition, leptin functions perinatally as a neurotrophic factor to facilitate the development of the neural circuits that govern metabolism, adipose tissue distribution and ingestive behavior (reviewed by Cottrell and Ozanne, 2007, 2008; Breton, 2013). In rats and mice, there is a surge in plasma leptin concentration during postnatal days (PND) 4–12 (Ahima et al., 1998; Delahaye et al., 2008; Cottrell et al., 2009), the time when connections between hypothalamic neurons are developing and leptin has no effect on appetite (Ahima and Flier, 2000). Rodents exposed to reduced nutrient availability have reduced circulating leptin concentrations and increased hypothalamic LepR protein expression at birth (Cottrell et al., 2010; Coupe et al., 2010), as well as a delayed and/or reduced leptin surge, which results in permanent changes in hypothalamic ingestive behavior regulatory circuits (Gluckman et al., 2005; Bautista et al., 2008). Recent studies provide further convincing evidence that leptin specifically promotes the development of Arc neuronal projections, consistent with a role in brain development. Arc projections in mice are formed primarily during the second week of postnatal life (reviewed by Ahima and Flier, 2000), developmentally similar to human third trimester of pregnancy. In leptin deficient (*ob/ob*) mice, these projection pathways regulating appetite are permanently disrupted, causing Arc axonal densities one third to one fourth that of controls (reviewed by Ahima and Flier, 2000). Reduced gestational nutrient availability permanently impairs the development and connectivity of hypothalamic neurons that produce anorectic neuropeptides such as α-MSH and orexigenic neuropeptides such as NPY and AgRP from weaning through adulthood (Muhlhausler et al., 2006; Delahaye et al., 2008; Coupe et al., 2010). Gestationally-restricted offspring also display resistance to the anorectic effects of peripheral leptin treatment in adulthood (reviewed by Breton, 2013). Therefore, leptin appears to be a critical factor for the gestational programming of appetite regulatory circuits as well.

Alterations in the Arc appetite regulatory network that increase production of and sensitivity to orexigenic agents such as NPY are clearly important for the programmed hyperphagia that occurs in response to perinatal nutritional perturbations. As mentioned above, the complex nature of ingestive behavior regulation makes is highly unlikely that just focusing on the Arc will be sufficient to explain all the effects of gestational nutrient restriction on appetite regulation. Alterations in dopamine expression in the VTA may also be critically important because the reward system is likely also to have gestationally programmed changes that may influence ingestive behavior regulation. For example, female LBW rats are more likely than non-restricted offspring to binge on sucrose when it is presented intermittently for limited amounts of time (unpublished observation). Therefore, further research into the contribution of reward circuitry to the mechanisms of gestational programming of appetite is clearly warranted.

A plethora of data supports the hypothesis that perinatal nutritional disturbances majorly contribute to the development of adult obesity by altering appetite regulation to favor hyperphagia. This complex system of neuroendocrine factors undergoes a short period of plasticity during development when the nutritional environment programs this system to maximize an organism's chances for survival in its anticipated postnatal environment. Several rodent models have been used to study the mechanisms responsible for perinatal appetite programming, including the maternal food restriction, low protein, lactational overnutrition, gestational diabetes, and high fat diet during pregnancy. Currently, there is not a validated model for the study of gestational programming on appetitive ingestive behaviors.

### Appetitive ingestive behaviors

Hamsters have several unique characteristics that make them ideal for studying the gestational programming of the regulation of appetitive ingestive behaviors, including the fact that hamsters utilize food hoarding as a significant part of their natural ingestive behavioral repertoire, and, unlike rats, hamsters do not overeat after energetic challenges such as food deprivation and pregnancy (Silverman and Zucker, 1976; Rowland, 1982; Billington et al., 1984; Bartness and Clein, 1994; Bartness et al., 1995). As mentioned above, the effects of gestational nutrient availability on appetitive ingestive behaviors have yet to be studied. Therefore, we recently endeavored to create a model of gestational programming in the hamster. In this model, pregnant hamsters are provided with daily food rations equal to pre-pregnancy intake, preventing maternal food hoarding, a behavior naturally enhanced during the last half of pregnancy. Interestingly, male offspring of restricted mothers exhibit hyperphagia and increased Arc NPY as well as decreased hoarding behavior (unpublished observations). Further characterization of this exciting new model of gestational programming should provide additional insights into the effects of prenatal environment on the neuroendocrine regulation of appetitive ingestive behaviors.

## Summary and conclusions

Obesity rates are on the rise worldwide, and research into the neuroendocrine regulation of appetitive ingestive behavior is likely to provide new information about how animals maintain energy balance in their native settings and how to combat the obesity epidemic. Fluctuating food supply has been a threat to survival and a determinant of reproductive success for most animals, including humans (Prentice, 2005; Schneider, 2006). Selection pressures resulting from frequent bouts of famine in the evolutionary history of most organisms favors a “thrifty,” a.k.a., metabolically efficient phenotype to increase the likelihood of reproductive success in an environment with a variable food supply (Prentice, 2005; Schneider, 2006). The overabundance of highly palatable, easily accessible food sources present in the human environment in developed countries has turned these adaptations into health liabilities. Unfortunately, the list of neuropeptides that “regulate” appetite is now overwhelming, and most ingestive behavior research continues to focus on the hypothalamic Arc nucleus as the command center for appetite regulation (reviewed by Barsh and Schwartz, 2002; Zigman and Elmquist, 2003; Schwartz and Porte, 2005).

Ingestive behavior regulation is far more complex than can be explained by studies conducted solely on the Arc nucleus. In addition, the study of the neuroendocrine regulation of appetitive behaviors provides clues to the mechanisms responsible for the motivation to eat. This review seeks to demonstrate that while hypothalamic appetite regulatory circuits are important for the control of both appetitive and consummatory ingestive behaviors, it is necessary to think more broadly than the “Arc perspective” by including systems such as the caudal hindbrain, the HPA axis and central reward circuitry to provide a more complete picture of neuroendocrine regulation of ingestive behavior.

Since the creation of the thrifty phenotype hypothesis by Neel in (1962), the maternal programming hypothesis of Jones et al. in (1984), and it's reiteration by Barker in (1994), understanding how nutrient availability during critical periods of development affects offspring appetite and energy balance has been a major focus of obesity research (Jones et al., 1984; Barker, 1994; Neel, 1999). A great deal of research has been done to characterize the effects of reduced perinatal nutrient availability on offspring consummatory ingestive behavior. Although research has not previously been conducted to determine how appetitive ingestive behaviors are impacted by maternal nutrient availability, future studies using hamsters as an animal model may provide critical information on the gestational programming of neuroendocrine systems that regulate feeding motivation.

### Conflict of interest statement

The authors declare that the research was conducted in the absence of any commercial or financial relationships that could be construed as a potential conflict of interest.

## References

[B1] Abizaid A. (2009). Ghrelin and dopamine: new insights on the peripheral regulation of appetite. J. Neuroendocrinol. 21, 787–793 10.1111/j.1365-2826.2009.01896.x 19523165

[B2] Abizaid A. Gao Q. Horvath T. L. (2006a). Thoughts for food: brain mechanisms and peripheral energy balance. Neuron 51, 691–702 10.1016/j.neuron.2006.08.025 16982416

[B3] Abizaid A. Liu Z. W. Andrews Z. B. Shanabrough M. Borok E. Elsworth J. D. (2006b). Ghrelin modulates the activity and synaptic input organization of midbrain dopamine neurons while promoting appetite. J. Clin. Invest. 116, 3229–3239 10.1172/JCI29867 17060947 PMC1618869

[B4] Abizaid A. Horvath T. L. (2012). Ghrelin and the central regulation of feeding and energy balance. Indian J. Endocrinol. Metab. 16, S617–S626 10.4103/2230-8210.105580 23565498 PMC3602992

[B5] Ahima R. S. Flier J. S. (2000). Adipose tissue as an endocrine organ. Trends Endocrinol. Metab. 11, 327–332 10.1016/S1043-2760(00)00301-5 10996528

[B6] Ahima R. S. Prabakaran D. Flier J. S. (1998). Postnatal leptin surge and regulation of circadian rhythm of leptin by feeding. Implications for energy homeostasis and neuroendocrine function. J. Clin. Invest. 101, 1020–1027 10.1172/JCI1176 9486972 PMC508653

[B7] Ariyasu H. Takaya K. Tagami T. Ogawa Y. Hosoda K. Akamizu T. (2001). Stomach is a major source of circulating ghrelin, and feeding state determines plasma ghrelin-like immunoreactivity levels in humans. J. Clin. Endocrinol. Metab. 86, 4753–4758 10.1210/jc.86.10.4753 11600536

[B8] Armitage J. A. Khan I. Y. Taylor P. D. Nathanielsz P. W. Poston L. (2004). Developmental programming of the metabolic syndrome by maternal nutritional imbalance: how strong is the evidence from experimental models in mammals? J. Physiol. 561, 355–377 10.1113/jphysiol.2004.072009 15459241 PMC1665360

[B9] Asakawa A. Inui A. Kaga T. Yuzuriha H. Nagata T. Ueno N. (2001). Ghrelin is an appetite-stimulatory signal from stomach with structural resemblance to motilin. Gastroenterology 120, 337–345 10.1053/gast.2001.22158 11159873

[B10] Baird J. Fisher D. Lucas P. Kleijnen J. Roberts H. Law C. (2005). Being big or growing fast: systematic review of size and growth in infancy and later obesity. BMJ 331, 929 10.1136/bmj.38586.411273.E0 16227306 PMC1261184

[B11] Ball G. F. Balthazart J. (2008). How useful is the appetitive and consummatory distinction for our understanding of the neuroendocrine control of sexual behavior? Horm. Behav. 53, 307–311 10.1016/j.yhbeh.2007.09.023 18045597 PMC3522858

[B12] Barker D. J. (1994). Outcome of low birthweight. Horm. Res. 42, 223–230 10.1159/000184197 7868077

[B13] Barsh G. S. Schwartz M. W. (2002). Genetic approaches to studying energy balance: perception and integration. Nat. Rev. Genet. 3, 589–600 10.1038/nrg862 12154382

[B14] Bartness T. J. (1997). Food hoarding is increased by pregnancy, lactation and food deprivation in Siberian hamsters. Am. J. Physiol. 272, R118–R125 9038999 10.1152/ajpregu.1997.272.1.R118

[B15] Bartness T. J. Clein M. R. (1994). Effects of food deprivation and restriction, and metabolic blockers on food hoarding in Siberian hamsters. Am. J. Physiol. 266, R1111–R1117 8184952 10.1152/ajpregu.1994.266.4.R1111

[B16] Bartness T. J. Keen-Rhinehart E. Dailey M. J. Teubner B. J. (2011). Neural and hormonal control of food hoarding. Am. J. Physiol. Regul. Integr. Comp. Physiol. 301, R641–R655 10.1152/ajpregu.00137.2011 21653877 PMC3290004

[B17] Bartness T. J. Morley J. E. Levine A. S. (1995). Effects of food deprivation and metabolic fuel utilization on the photoperiodic control of food intake in Siberian hamsters. Physiol. Behav. 57, 61–68 10.1016/0031-9384(94)00203-H 7710560

[B18] Baskin D. G. Blevins J. E. Schwartz M. W. (2001). How the brain regulates food intake and body weight: the role of leptin. J Pediatr. Endocrinol. Metab. 14(Suppl. 6), 1417–1429 11837495

[B19] Baskin D. G. Hahn T. M. Schwartz M. W. (1999). Leptin sensitive neurons in the hypothalamus. Horm. Metab. Res. 31, 345–350 10.1055/s-2007-978751 10422733

[B20] Bautista C. J. Boeck L. Larrea F. Nathanielsz P. W. Zambrano E. (2008). Effects of a maternal low protein isocaloric diet on milk leptin and progeny serum leptin concentration and appetitive behavior in the first 21 days of neonatal life in the rat. Pediatr. Res. 63, 358–363 10.1203/01.pdr.0000304938.78998.21 18356739

[B21] Beneke W. M. Davis C. H. (1985). Relationship of hunger, use of a shopping list and obesity to food purchases. Int. J. Obes. 9, 391–399 3830932

[B22] Bergonzelli G. E. Pralong F. P. Glauser M. Cavadas C. Grouzmann E. Gaillard R. C. (2001). Interplay between galanin and leptin in the hypothalamic control of feeding via corticotropin-releasing hormone and neuropeptide Y. Diabetes 50, 2666–2672 10.2337/diabetes.50.12.2666 11723048

[B23] Billington C. J. Morley J. E. Levine A. S. Gerritsen G. C. (1984). Feeding systems in Chinese hamsters. Am. J. Physiol. 247, R405–R411 6148021 10.1152/ajpregu.1984.247.3.R405

[B24] Brady L. S. Smith M. A. Gold P. W. Herkenham M. (1990). Altered expression of hypothalamic neuropeptide mRNAs in food-restricted and food-deprived rats. Neuroendocrinology 52, 441–447 10.1159/000125626 2177853

[B25] Bray G. A. Stern J. S. Castonguay T. W. (1992). Effect of adrenalectomy and high-fat diet on the fatty Zucker rat. Am. J. Physiol. 262, E32–E39 1733248 10.1152/ajpendo.1992.262.1.E32

[B26] Breton C. (2013). The hypothalamus-adipose axis is a key target of developmental programming by maternal nutritional manipulation. J. Endocrinol. 216, R19–R31 10.1530/JOE-12-0157 23108716

[B144] Briggs D. I. Andrews Z. B. (2011). Metabolic status regulates ghrelin function on energy homeostasis. Neuroendocrinology 93, 48–57 10.1159/000322589 21124019

[B27] Bronson F. H. (1989). Mammalian Reproductive Biology, Chicago: University of Chicago Press

[B28] Bruce B. K. King B. M. Phelps G. R. Veitia M. C. (1982). Effects of adrenalectomy and corticosterone administration on hypothalamic obesity in rats. Am. J. Physiol. 243, E152–E157 7202335 10.1152/ajpendo.1982.243.2.E152

[B29] Buckley C. A. Schneider J. E. (2003). Food hoarding is increased by food deprivation and decreased by leptin treatment in Syrian hamsters. Am. J. Physiol. Regul. Integr. Comp. Physiol. 285, R1021–R1029 10.1152/ajpregu.00488.2002 12730075

[B30] Caballero B. (2007). The global epidemic of obesity: an overview. Epidemiol. Rev. 29, 1–5 10.1093/epirev/mxm012 17569676

[B31] Cabanac M. Dagnault A. Richard D. (1997). The food-hoarding threshold is not raised by acute intraventricular NPY in male rats. Physiol. Behav. 61, 131–135 10.1016/S0031-9384(97)00349-1 8976543

[B32] Cabanac M. Richard D. (1995). Acute intraventricular CRF lowers the hoarding threshold in male rats. Physiol. Behav. 57, 705–710 10.1016/0031-9384(94)00322-X 7777607

[B33] Carr J. A. (2002). Stress, neuropeptides, and feeding behavior: a comparative perspective. Integr. Comp. Biol. 42, 582–590 10.1093/icb/42.3.582 21708754

[B34] Carr J. A. Brown C. L. Mansouri R. Venkatesan S. (2002). Neuropeptides and amphibian prey-catching behavior. Comp. Biochem. Physiol. B Biochem. Mol. Biol. 132, 151–162 10.1016/S1096-4959(01)00545-0 11997218

[B35] Chen H. Y. Trumbauer M. E. Chen A. S. Weingarth D. T. Adams J. R. Frazier E. G. (2004). Orexigenic action of peripheral ghrelin is mediated by neuropeptide Y and agouti-related protein. Endocrinology 145, 2607–2612 10.1210/en.2003-1596 14962995

[B36] Cone R. D. Cowley M. A. Butler A. A. Fan W. Marks D. L. Low M. J. (2001). The arcuate nucleus as a conduit for diverse signals relevant to energy homeostasis. Int. J. Obes. Relat. Metab. Disord. 25(Suppl. 5), S63–S67 10.1038/sj.ijo.0801913 11840218

[B37] Coppari R. Ichinose M. Lee C. E. Pullen A. E. Kenny C. D. McGovern R. A. (2005). The hypothalamic arcuate nucleus: a key site for mediating leptin's effects on glucose homeostasis and locomotor activity. Cell Metab. 1, 63–72 10.1016/j.cmet.2004.12.004 16054045

[B38] Cortelazzi D. Cappiello V. Morpurgo P. S. Ronzoni S. Nobile De Santis M. S. Cetin I. (2003). Circulating levels of ghrelin in human fetuses. Eur. J. Endocrinol. 149, 111–116 10.1530/eje.0.1490111 12887287

[B39] Cottrell E. C. Cripps R. L. Duncan J. S. Barrett P. Mercer J. G. Herwig A. (2009). Developmental changes in hypothalamic leptin receptor: relationship with the postnatal leptin surge and energy balance neuropeptides in the postnatal rat. Am. J. Physiol. Regul. Integr. Comp. Physiol. 296, R631–R639 10.1152/ajpregu.90690.2008 19144754 PMC2665846

[B40] Cottrell E. C. Mercer J. G. Ozanne S. E. (2010). Postnatal development of hypothalamic leptin receptors. Vitam. Horm. 82, 201–217 10.1016/S0083-6729(10)82011-4 20472140

[B41] Cottrell E. C. Ozanne S. E. (2007). Developmental programming of energy balance and the metabolic syndrome. Proc. Nutr. Soc. 66, 198–206 10.1017/S0029665107005447 17466102

[B42] Cottrell E. C. Ozanne S. E. (2008). Early life programming of obesity and metabolic disease. Physiol. Behav. 94, 17–28 10.1016/j.physbeh.2007.11.017 18155097

[B43] Coupe B. Amarger V. Grit I. Benani A. Parnet P. (2010). Nutritional programming affects hypothalamic organization and early response to leptin. Endocrinology 151, 702–713 10.1210/en.2009-0893 20016030

[B44] Craig W. (1918). Appetites and aversions as constituents of instincts. Biol. Bull. 34, 91–107 10.2307/1536346 16586767 PMC1091358

[B45] Cripps R. L. Martin-Gronert M. S. Ozanne S. E. (2005). Fetal and perinatal programming of appetite. Clin. Sci. (Lond) 109, 1–11 10.1042/CS20040367 15966867

[B46] Dailey M. J. Bartness T. J. (2010). Arcuate nucleus destruction does not block food deprivation-induced increases in food foraging and hoarding. Brain Res. 1323, 94–108 10.1016/j.brainres.2010.01.078 20138163 PMC2896996

[B47] Dallman M. F. la Fleur S. E. Pecoraro N. C. Gomez F. Houshyar H. Akana S. F. (2004). Minireivew: glucocorticoids – food intake, abdominal obesity and wealthy nations in 2004. Endocrinology 145, 2633–2638 10.1210/en.2004-0037 15044359

[B48] Dallman M. F. Strack A. M. Akana S. F. Bradbury M. J. Hanson E. S. Scribner K. A. (1993). Feast and famine: critical role of glucocorticoids with insulin in daily energy flow. Front. Neuroendocinol. 14, 303–347 10.1006/frne.1993.1010 8258378

[B49] Day D. E. Bartness T. J. (2003). Fasting-induced increases in hoarding are dependent on the foraging effort level. Physiol. Behav. 78, 655–668 10.1016/S0031-9384(03)00052-0 12782221

[B50] Day D. E. Bartness T. J. (2004). Agouti-related protein increases food hoarding, but not food intake by Siberian hamsters. Am. J. Physiol. 286, R38–R45 10.1152/ajpregu.00284.2003 14500267

[B51] Day D. E. Keen-Rhinehart E. Bartness T. J. (2005). Role of NPY and its receptor subtypes in foraging, food hoarding and food intake by Siberian hamsters. Am. J. Physiol. 289, R29–R36 10.1152/ajpregu.00853.2004 15705801

[B52] Delahaye F. Breton C. Risold P. Y. Enache M. Dutriez-Casteloot I. Laborie C. (2008). Maternal perinatal undernutrition drastically reduces postnatal leptin surge and affects the development of arcuate nucleus proopiomelanocortin neurons in neonatal male rat pups. Endocrinology 149, 470–475 10.1210/en.2007-1263 18006626

[B53] Dhillon H. Zigman J. M. Ye C. Lee C. E. McGovern R. A. Tang V. (2006). Leptin directly activates SF1 neurons in the VMH, and this action by leptin is required for normal body-weight homeostasis. Neuron 49, 191–203 10.1016/j.neuron.2005.12.021 16423694

[B54] Dodd D. K. Stalling R. B. Bedell J. (1977). Grocery purchases as a function of obesity and assumed food deprivation. Int. J. Obes. 1, 43–47 617107

[B145] Eckel L. A. (2011). The ovarian hormone estradiol plays a crucial role in the control of food intake in females. Physiol. Behav. 104, 517–524 10.1016/j.physbeh.2011.04.014 21530561 PMC3139826

[B55] Ellacott K. L. Cone R. D. (2004). The central melanocortin system and the integration of short- and long-term regulators of energy homeostasis. Recent Prog. Horm. Res. 59, 395–408 10.1210/rp.59.1.395 14749511

[B56] Elmquist J. K. (2001). Hypothalamic pathways underlying the endocrine, autonomic, and behavioral effects of leptin. Int. J. Obes. Relat. Metab. Disord. 25(Suppl. 5), S78–S82 10.1038/sj.ijo.0801918 11840221

[B57] Elmquist J. K. Coppari R. Balthasar N. Ichinose M. Lowell B. B. (2005). Identifying hypothalamic pathways controlling food intake, body weight, and glucose homeostasis. J. Comp. Neurol. 493, 63–71 10.1002/cne.20786 16254991

[B58] Epstein L. H. Dearing K. K. Paluch R. A. Roemmich J. N. Cho D. (2007). Price and maternal obesity influence purchasing of low- and high-energy-dense foods. Am. J. Clin. Nutr. 86, 914–922 17921365 10.1093/ajcn/86.4.914 PMC2175079

[B59] Everitt B. J. Hokfelt T. Terenius L. Tatemoto K. Mutt V. Goldstein M. (1984). Differential co-existence of neuropeptide Y (NPY)-like immunoreactivity with catecholamines in the central nervous system of the rat. Neuroscience 11, 443–462 10.1016/0306-4522(84)90036-8 6144080

[B60] Fantino M. Cabanac M. (1984). Effect of a cold ambient temperature on the rat's food hoarding behavior. Physiol. Behav. 32, 183–190 10.1016/0031-9384(84)90127-6 6538979

[B61] Faulconbridge L. F. Grill H. J. Kaplan J. M. (2005). Distinct forebrain and caudal brainstem contributions to the neuropeptide Y mediation of ghrelin hyperphagia. Diabetes 54, 1985–1993 10.2337/diabetes.54.7.1985 15983198

[B62] Feldkircher K. M. Mistry A. M. Romsos D. R. (1996). Adrenalectomy reverses pre-existing obesity in adult genetically obese (*ob/ob*) mice. Int. J. Obes. Relat. Metab. Disord. 20, 232–235 8653144

[B63] Fulton S. Pissios P. Manchon R. P. Stiles L. Frank L. Pothos E. N. (2006). Leptin regulation of the mesoaccumbens dopamine pathway. Neuron 51, 811–822 10.1016/j.neuron.2006.09.006 16982425

[B64] Fulton S. Richard D. Woodside B. Shizgal P. (2002). Interaction of CRH and energy balance in the modulation of brain stimulation reward. Behav. Neurosci. 116, 651–659 10.1037/0735-7044.116.4.651 12148932

[B142] Gao Q. Mezei G. Nie Y. Choi C. S. Bechmann I. Leranth C. (2007). Anorectic estrogen mimics leptin's effect on the rewiring of melanocortins cells and Stat3 signaling in obese animals. Nat. Med. 13, 89–94 10.1038/nm1525 17195839

[B65] Glowa J. R. Barrett J. E. Russell J. Gold P. W. (1992). Effects of corticotropin releasing hormone on appetitive behaviors. Peptides 13, 609–621 10.1016/0196-9781(92)90097-M 1523173

[B66] Gluckman P. D. Hanson M. A. Pinal C. (2005). The developmental origins of adult disease. Matern. Child Nutr. 1, 130–141 10.1111/j.1740-8709.2005.00020.x 16881892 PMC6860944

[B67] Gonzalez-Bulnes A. Ovilo C. (2012). Genetic basis, nutritional challenges and adaptive responses in the prenatal origin of obesity and type-2 diabetes. Curr. Diabetes Rev. 8, 144–154 10.2174/157339912799424537 22283677

[B68] Gosselin C. Cabanac M. (1997). Adrenalectomy lowers the body weight set-point in rats. Physiol. Behav. 62, 519–523 10.1016/S0031-9384(97)00010-3 9272658

[B69] Grill H. J. (2010). Leptin and the systems neuroscience of meal size control. Front. Neuroendocrinol. 31, 61–78 10.1016/j.yfrne.2009.10.005 19836413 PMC2813996

[B147] Grill H. J. Hayes M. R. (2009). The nucleus tractus solitarius: a portal for visceral afferent signal processing, energy status assessment and integration of their combined effects on food intake. Int. J. Obes. (Lond) 33, S11–S15 10.1038/ijo.2009.10 19363500

[B146] Grill H. J. Kaplan J. M. (1992). Sham feeding in intact and chronic decerebrate rats. Am. J. Physiol. 262, R1070–R1074 1621860 10.1152/ajpregu.1992.262.6.R1070

[B70] Grill H. J. Kaplan J. M. (2001). Interoceptive and integrative contributions of forebrain and brainstem to energy balance control. Int. J. Obes. Relat. Metab. Disord. 25(Suppl. 5), S73–S77 10.1038/sj.ijo.0801917 11840220

[B71] Grill H. J. Kaplan J. M. (2002). The neuroanatomical axis for control of energy balance. Front. Neuroendocrinol. 23, 2–40 10.1006/frne.2001.0224 11906202

[B72] Grill H. J. Smith G. P. (1988). Cholecystokinin decreases sucrose intake in chronic decerebrate rats. Am. J. Physiol. 254, R853–R856 3381910 10.1152/ajpregu.1988.254.6.R853

[B143] Gyengesi E. Liu W. D'Agostino G. Gan G. Horvath T. L. Gao X. B. (2010). Corticosterone regulates synaptic input organization of POMC and NPY/AgRP neurons in adult mice. Endocrinology 151, 5395–5402 10.1210/en.2010-0681 20843996 PMC2954711

[B73] Hahn T. M. Breininger J. F. Baskin D. G. Schwartz M. W. (1998). Coexpression of Agrp and NPY in fasting-activated hypothalamic neurons. Nat. Neurosci. 1, 271–272 10.1038/1082 10195157

[B74] Hales C. N. Barker D. J. (2001). The thrifty phenotype hypothesis. Br. Med. Bull. 60, 5–20 10.1093/bmb/60.1.5 11809615

[B75] Harder T. Bergmann R. Kallischnigg G. Plagemann A. (2005). Duration of breastfeeding and risk of overweight: a meta-analysis. Am. J. Epidemiol. 162, 397–403 10.1093/aje/kwi222 16076830

[B76] Haslam D. (2007). Obesity: a medical history. Obes. Rev. 8(Suppl. 1), 31–36 10.1111/j.1467-789X.2007.00314.x 17316298

[B77] Heinrichs S. C. Richard D. (1999). The role of corticotropin-releasing factor and urocortin in the modulation of ingestive behavior. Neuropeptides 33, 350–359 10.1054/npep.1999.0047 10657512

[B78] Hill J. O. Fried S. K. DiGirolamo M. (1983). Effects of a high-fat diet on energy intake and expenditure in rats. Life Sci. 33, 141–149 10.1016/0024-3205(83)90406-X 6865651

[B79] Hill J. O. Fried S. K. DiGirolamo M. (1984). Effects of fasting and restricted refeeding on utilization of ingested energy in rats. Am. J. Physiol. 247, R318–R327 6465347 10.1152/ajpregu.1984.247.2.R318

[B80] Hirasawa M. Parsons M. P. Alberto C. O. (2007). Interaction between orexins and the mesolimbic system for overriding satiety. Rev. Neurosci. 18, 383–393 10.1515/REVNEURO.2007.18.5.383 19544624

[B81] Hommel J. D. Trinko R. Sears R. M. Georgescu D. Liu Z. W. Gao X. B. (2006). Leptin receptor signaling in midbrain dopamine neurons regulates feeding. Neuron 51, 801–810 10.1016/j.neuron.2006.08.023 16982424

[B82] Jones A. P. Simson E. L. Friedman M. I. (1984). Gestational undernutrition and the development of obesity in rats. J. Nutr. 114, 1484–1492 6540299 10.1093/jn/114.8.1484

[B83] Kageyama H. Takenoya F. Hirako S. Wada N. Kintaka Y. Inoue S. (2012). Neuronal circuits involving neuropeptide Y in hypothalamic arcuate nucleus-mediated feeding regulation. Neuropeptides 46, 285–289 10.1016/j.npep.2012.09.007 23110814

[B84] Kalra S. P. Dube M. G. Shuye P. Bin X. Horvath T. L. Kalra P. S. (1999). Interacting appetite-regulating pathways in the hypothalamic regulation of body weight. Endocrine Rev. 20, 68–100 10.1210/er.20.1.68 10047974

[B85] Karolyi I. J. Burrows H. L. Ramesh T. M. Nakajima M. Lesh J. S. Seong E. (1999). Altered anxiety and weight gain in corticotropin-releasing hormone-binding protein-deficient mice. Proc. Natl. Acad. Sci. U.S.A. 96, 11595–11600 10.1073/pnas.96.20.11595 10500222 PMC18079

[B86] Keen-Rhinehart E. Bartness T. J. (2005). Peripheral ghrelin injections stimulate food intake, foraging and food hoarding in Siberian hamsters. Am. J. Physiol. 288, R716–R722 10.1152/ajpregu.00705.2004 15576659

[B87] Keen-Rhinehart E. Bartness T. J. (2007). NPY Y1 receptor is involved in ghrelin- and fasting-induced increases in foraging, food hoarding, and food intake. Am. J. Physiol. Regul. Integr. Comp. Physiol. 292, R1728–R1737 10.1152/ajpregu.00597.2006 17204592 PMC3509278

[B88] Keen-Rhinehart E. Bartness T. J. (2008). Leptin inhibits food-deprivation-induced increases in food intake and food hoarding. Am. J. Physiol. Regul. Integr. Comp. Physiol. 295, R1737–R1746 10.1152/ajpregu.90512.2008 18832088 PMC3509279

[B89] Keen-Rhinehart E. Dailey M. J. Bartness T. (2010). Physiological mechanisms for food-hoarding motivation in animals. Philos. Trans. R. Soc. Lond. B Biol. Sci. 365, 961–975 10.1098/rstb.2009.0225 20156819 PMC2830250

[B90] Landys M. M. Ramenofsky M. Wingfield J. C. (2006). Actions of glucocorticoids at a seasonal baseline as compared to stress-related levels in the regulation of periodic life processes. Gen. Comp. Endocrinol. 148, 132–149 10.1016/j.ygcen.2006.02.013 16624311

[B91] Lyons A. M. Thiele T. E. (2010). Neuropeptide Y conjugated to saporin alters anxiety-like behavior when injected into the central nucleus of the amygdala or basomedial hypothalamus in BALB/cJ mice. Peptides 31, 2193–2199 10.1016/j.peptides.2010.09.009 20863864 PMC2971693

[B92] Mela D. J. Aaron J. I. Gatenby S. J. (1996). Relationships of consumer characteristics and food deprivation to food purchasing behavior. Physiol. Behav. 60, 1331–1335 10.1016/S0031-9384(96)00241-7 8916190

[B93] Mercer J. G. Moar K. M. Ross A. W. Hoggard N. Morgan P. J. (2000). Photoperiod regulates arcuate nucleus POMC, AGRP, and leptin receptor mRNA in Siberian hamster hypothalamus. Am. J. Physiol. 278, R271–R281 10.1006/appe.1999.0301 10644649

[B94] Michel C. Cabanac M. (1999). Lipectomy, body weight, and body weight set point in rats. Physiol. Behav. 66, 473–479 10.1016/S0031-9384(98)00317-5 10357436

[B95] Morton G. J. Schwartz M. W. (2001). The NPY/AgRP neuron and energy homeostasis. Int. J. Obes. Relat. Metab. Disord. 25(Suppl. 5), S56–S62 10.1038/sj.ijo.0801915 11840217

[B96] Muhlhausler B. S. (2007). Programming of the appetite-regulating neural network: a link between maternal overnutrition and the programming of obesity? J. Neuroendocrinol. 19, 67–72 10.1111/j.1365-2826.2006.01505.x 17184487

[B97] Muhlhausler B. S. Adam C. L. Findlay P. A. Duffield J. A. McMillen I. C. (2006). Increased maternal nutrition alters development of the appetite-regulating network in the brain. FASEB J. 20, 1257–1259 10.1096/fj.05-5241fje 16684802

[B98] Musatov S. Chen W. Pfaff D. W. Mobbs C. V. Yang X. J. Clegg D. J. (2007). Silencing of estrogen receptor alpha in the ventromedial nucleus of hypothalamus leads to metabolic syndrome. Proc. Natl. Acad. Sci. U.S.A. 104, 2501–2506 10.1073/pnas.0610787104 17284595 PMC1892990

[B99] Nagata E. Nakagawa Y. Yamaguchi R. Fujisawa Y. Sano S. Satake E. (2011). Altered gene expressions of ghrelin, PYY, and CCK in the gastrointestinal tract of the hyperphagic intrauterine growth restriction rat offspring. Horm. Metab. Res. 43, 178–182 10.1055/s-0030-1270528 21264794

[B149] Neel J. V. (1962). Diabetes mellitus: a “thrifty” genotype rendered detrimental by “progress”? Am. J. Hum. Genet. 14, 353–362 13937884 PMC1932342

[B100] Neel J. V. (1999). Diabetes mellitus: a “thrifty” genotype rendered detrimental by “progress”? 1962. Bull. World Health Organ. 77, 694–703 10516792 PMC2557712

[B101] Pandit R. de Jong J. W. Vanderschuren L. J. Adan R. A. (2011). Neurobiology of overeating and obesity: the role of melanocortins and beyond. Eur. J. Pharmacol. 660, 28–42 10.1016/j.ejphar.2011.01.034 21295024

[B102] Prentice A. M. (2005). Early influences on human energy regulation: thrifty genotypes and thrifty phenotypes. Physiol. Behav. 86, 640–645 10.1016/j.physbeh.2005.08.055 16260008

[B103] Ransley J. K. Donnelly J. K. Botham H. Khara T. N. Greenwood D. C. Cade J. E. (2003). Use of supermarket receipts to estimate energy and fat content of food purchased by lean and overweight families. Appetite 41, 141–148 10.1016/S0195-6663(03)00051-5 14550311

[B104] Romsos D. R. Vander Tuig J. G. Kerner J. Grogan C. K. (1987). Energy balance in rats with obesity-producing hypothalamic knife cuts: effects of adrenalectomy. J. Nutr. 117, 1121–1128 3598723 10.1093/jn/117.6.1121

[B105] Ross I. Smith W. I. (1953). The hoarding behavior of the mouse II. The role of deprivation, satiation and stress. J. Gen. Psychol. 82, 279–297 13096723 10.1080/08856559.1953.10534495

[B106] Rowland N. E. (1982). Failure by deprived hamsters to increase food intake: some behavioral and physiological determinants. J. Comp. Physiol. Psychol. 96, 591–603 10.1037/h0077905 7119177

[B107] Sahin H. Erener T. Erginoz E. Vural M. Ilikkan B. Kavuncuoglu S. (2012). The relationship of active ghrelin levels and intrauterine growth in preterm infants. Eur. J. Endocrinol. 166, 399–405 10.1530/EJE-11-0607 22143318

[B108] Sapolsky R. M. Romero L. M. Munck A. U. (2000). How do glucocorticoids influence stress responses? Integrating permissive, suppressive, stimulatory, and preparative actions. Endocr. Rev. 21, 55–89 10.1210/er.21.1.55 10696570

[B109] Schellekens H. Finger B. C. Dinan T. G. Cryan J. F. (2012). Ghrelin signalling and obesity: at the interface of stress, mood and food reward. Pharmacol. Ther. 135, 316–326 10.1016/j.pharmthera.2012.06.004 22749794

[B110] Schneider J. E. (2006). Metabolic and hormonal control of the desire for food and sex: implications for obesity and eating disorders. Horm. Behav. 50, 562–571 10.1016/j.yhbeh.2006.06.023 16875692

[B111] Schneider J. E. Klingerman C. M. Abdulhay A. (2012). Sense and nonsense in metabolic control of reproduction. Front. Endocrinol. (Lausanne) 3:26 10.3389/fendo.2012.00026 22649413 PMC3355988

[B112] Schneider J. E. Wade G. N. (1999). Inhibition of reproduction in service of energy balance, in Reproduction in Context: Environmental and Social Influences on Reproductive Physiology and Behavior, eds Wallen K. Schneider J. E. (Cambridge, MIT Press), 35–82

[B113] Schneider J. E. Watts A. G. (2002). Energy balance, ingestive behavior and reproductive success, in Hormones Brain and Behavior, ed D. Pfaff (San Diego, CA: Academic Press), 435–523 10.1016/B978-012532104-4/50009-3

[B148] Schneider J. E. Buckley C. A. Blum R. M. Zhou D. Szymanski L. Day D. E. (2002). Metabolic signals, hormones and neuropeptides involved in control of energy balance and reproductive success in hamsters. Eur. J. Neurosci. 16, 377–379 10.1046/j.1460-9568.2002.02118.x 12193177

[B114] Schneider J. E. Wise J. D. Benton N. A. Brozek J. M. Keen-Rhinehart E. (2013). When do we eat? Ingestive behavior, survival, and reproductive success. Horm. Behav. 64, 702–728 10.1016/j.yhbeh.2013.07.005 23911282

[B115] Schwartz M. W. Porte D. Jr. (2005). Diabetes, obesity, and the brain. Science 307, 375–379 10.1126/science.1104344 15662002

[B116] Shankar K. Harrell A. Liu X. Gilchrist J. M. Ronis M. J. Badger T. M. (2008). Maternal obesity at conception programs obesity in the offspring. Am. J. Physiol. Regul. Integr. Comp. Physiol. 294, R528–R538 10.1152/ajpregu.00316.2007 18032473

[B117] Silverman H. J. Zucker I. (1976). Absence of post-fast food compensation in the golden hamster (Mesocricetus auratus). Physiol. Behav. 17, 271–285 10.1016/0031-9384(76)90076-7 1033582

[B118] Sonneville K. R. Rifas-Shiman S. L. Haines J. Gortmaker S. Mitchell K. F. Gillman M. W. (2013). Associations of parental control of feeding with eating in the absence of hunger and food sneaking, hiding, and hoarding. Child Obes. 9, 346–349 10.1089/chi.2012.0149 23806073 PMC3728724

[B119] Stanley B. G. Lanthier D. Chin A. S. Leibowitz S. F. (1989). Suppression of neuropeptide Y-elicited eating by adrenalectomy or hypophysectomy: reversal with corticosterone. Brain Res. 501, 32–36 10.1016/0006-8993(89)91023-8 2804697

[B120] Stevenson J. A. Franklin C. (1970). Effects of ACTH and corticosteroids in the regulation of food and water intake. Prog. Brain Res. 32, 141–152 10.1016/S0079-6123(08)61529-0 5491100

[B121] Stice E. Figlewicz D. P. Gosnell B. A. Levine A. S. Pratt W. E. (2012). The contribution of brain reward circuits to the obesity epidemic. Neurosci. Biobehav. Rev. [Epub ahead of print]. 10.1016/j.neubiorev.2012.12.001 23237885 PMC3604128

[B122] Teubner B. J. Bartness T. J. (2010). Cholecystokinin-33 acutely attenuates food foraging, hoarding and intake in Siberian hamsters. Peptides 31, 618–624 10.1016/j.peptides.2009.12.010 20025915 PMC2837760

[B123] Teubner B. J. Bartness T. J. (2013). Anti-ghrelin spiegelmer inhibits exogenous ghrelin-induced increases in food intake, hoarding and neural activation, but not food deprivation-induced increases. Am. J. Physiol. Regul. Integr. Comp. Physiol. 305, R323–R333 10.1152/ajpregu.00097.2013 23804279 PMC3833397

[B124] Teubner B. J. Garretson J. T. Hwang Y. Cole P. A. Bartness T. J. (2013). Inhibition of ghrelin O-acyltransferase attenuates food deprivation-induced increases in ingestive behavior. Horm. Behav. 63, 667–673 10.1016/j.yhbeh.2013.02.001 23399323 PMC3633643

[B125] Teubner B. J. Keen-Rhinehart E. Bartness T. J. (2012). Third ventricular coinjection of subthreshold doses of NPY and AgRP stimulate food hoarding and intake and neural activation. Am. J. Physiol. Regul. Integr. Comp. Physiol. 302, R37–R48 10.1152/ajpregu.00475.2011 22012701 PMC3349384

[B126] Tom G. (1983). Effect of deprivation on the grocery shopping behavior of obese and nonobese consumers. Int. J. Obes. 7, 307–311 6629638

[B127] Ulijaszek S. J. (2002). Human eating behaviour in an evolutionary ecological context. Proc. Nutr. Soc. 61, 517–526 10.1079/PNS2002180 12691181

[B128] van Dijk G. Donahey J. C. K. Thiele T. E. Scheurink A. J. W. Steffens A. B. Wilkinson C. W. (1997). Central leptin stimulates corticosterone secretion at the onset of the dark phase. Diabetes 46, 1911–1914 10.2337/diab.46.11.1911 9356047

[B129] Vickers M. H. (2007). Developmental programming and adult obesity: the role of leptin. Curr. Opin. Endocrinol. Diabetes Obes. 14, 17–22 10.1097/MED.0b013e328013da48 17940414

[B130] Vickers M. H. Krechowec S. O. Breier B. H. (2007). Is later obesity programmed *in utero*? Curr. Drug Targets 8, 923–934 10.2174/138945007781386857 17691929

[B131] Volkow N. D. Wang G. J. Tomasi D. Baler R. D. (2013). Obesity and addiction: neurobiological overlaps. Obes. Rev. 14, 2–18 10.1111/j.1467-789X.2012.01031.x 23016694 PMC4827343

[B132] Wade G. N. (1976). Sex hormones, regulatory behaviors, and body weight, in Advances in the Study of Behavior, Vol. 6, eds Rosenblatt J. S. Hinde R. A. Shaw E. Beer C. G. (New York, NY: Academic Press), 201–279

[B133] Wang X. Liang L. Du L. (2007). The effects of intrauterine undernutrition on pancreas ghrelin and insulin expression in neonate rats. J. Endocrinol. 194, 121–129 10.1677/JOE-07-0057 17592026

[B134] Wood A. D. Bartness T. J. (1996a). Caloric density affects food hoarding and intake by Siberian hamsters. Physiol. Behav. 59, 897–903 10.1016/0031-9384(95)02167-1 8778884

[B135] Wood A. D. Bartness T. J. (1996b). Food deprivation-induced increases in hoarding by Siberian hamsters are not photoperiod-dependent. Physiol. Behav. 60, 1137–1145 10.1016/0031-9384(96)00173-4 8884944

[B136] Wu Q. Boyle M. P. Palmiter R. D. (2009). Loss of GABAergic signaling by AgRP neurons to the parabrachial nucleus leads to starvation. Cell 137, 1225–1234 10.1016/j.cell.2009.04.022 19563755 PMC2729323

[B137] Yang H. D. Wang Q. Wang Z. Wang D. H. (2011). Food hoarding and associated neuronal activation in brain reward circuitry in Mongolian gerbils. Physiol. Behav. 104, 429–436 10.1016/j.physbeh.2011.04.062 21570992

[B138] Yousheng J. Nguyen T. Desai M. Ross M. G. (2008). Programmed alterations in hypothalamic neuronal orexigenic responses to ghrelin following gestational nutrient restriction. Reprod. Sci. 15, 702–709 10.1177/1933719108316982 18562700

[B139] Zhang X. Y. Yang H. D. Zhang Q. Wang Z. Wang D. H. (2011). Increased feeding and food hoarding following food deprivation are associated with activation of dopamine and orexin neurons in male Brandt's voles. PLoS ONE 6:e26408 10.1371/journal.pone.0026408 22046281 PMC3203142

[B140] Zheng H. Patterson L. M. Rhodes C. J. Louis G. W. Skibicka K. P. Grill H. J. (2010). A potential role for hypothalamomedullary POMC projections in leptin-induced suppression of food intake. Am. J. Physiol. Regul. Integr. Comp. Physiol. 298, R720–R728 10.1152/ajpregu.00619.2009 20071607 PMC2838656

[B141] Zigman J. M. Elmquist J. K. (2003). Minireview: from anorexia to obesity–the yin and yang of body weight control. Endocrinology 144, 3749–3756 10.1210/en.2003-0241 12933644

